# The Role of Glucose-Dependent Insulinotropic Polypeptide (GIP) in Bone Metabolism

**DOI:** 10.3390/ijms27020600

**Published:** 2026-01-07

**Authors:** Angyi Lin, Hideki Kitaura, Fumitoshi Ohori, Aseel Marahleh, Jinghan Ma, Ziqiu Fan, Kohei Narita, Kou Murakami, Hiroyasu Kanetaka

**Affiliations:** 1 Division of Orthodontics and Dentofacial Orthopedics, Tohoku University Graduate School of Dentistry, 4-1 Seiryo-Machi, Aoba-ku, Sendai 980-8575, Miyagi, Japan; lin.angyi.r5@dc.tohoku.ac.jp (A.L.); fumitoshi.ohori.b4@tohoku.ac.jp (F.O.); ma.jinghan.c1@tohoku.ac.jp (J.M.); fan.ziqiu.q1@dc.tohoku.ac.jp (Z.F.); kohei.narita.a2@tohoku.ac.jp (K.N.); kou.murakami.b2@tohoku.ac.jp (K.M.); hiroyasu.kanetaka.e6@tohoku.ac.jp (H.K.); 2 Creative Interdisciplinary Research Division, Frontier Research Institute for Interdisciplinary Sciences, Tohoku University, 6-3 Aramaki Aza Aoba, Aoba-ku, Sendai 980-8578, Miyagi, Japan; aseel.mahmoud.suleiman.marahleh.e6@tohoku.ac.jp; 3 Division of Advanced Dental Science and Technology, Graduate School of Biomedical Engineering, Tohoku University, 6-6-12, Aramaki Aza Aoba, Aoba-ku, Sendai 980-8579, Miyagi, Japan; 4 Division of Interdisciplinary Co-Creation (ICC-Division), Liaison Center for Innovative Dentistry, Tohoku University Graduate School of Dentistry, 4-1 Seiryo-Machi, Aoba-ku, Sendai 980-8575, Miyagi, Japan

**Keywords:** GIP, bone, CTX, P1NP, gut–bone axis

## Abstract

Glucose-dependent insulinotropic polypeptide (GIP) was the first incretin hormone identified, best known for promoting glucose-stimulated insulin secretion. Increasing evidence has expanded its physiological relevance beyond glucose metabolism, revealing a significant role for GIP in the gut–bone axis. In vitro studies demonstrate that GIP inhibits osteoclast differentiation and activity while promoting osteoblastic bone formation. Findings from genetic animal models and human variant analyses further support the essential role of endogenous GIP signaling in maintaining bone mass and quality. Exogenous administration of GIP suppresses the bone-resorption marker C-terminal telopeptide of type I collagen (CTX) and increases the bone-formation marker procollagen type I N-terminal propeptide (P1NP) in healthy individuals, reflecting an acute shift toward reduced bone resorption and enhanced bone formation. Moreover, GIP confers protection against bone deterioration in multiple pathological conditions, including postmenopausal osteoporosis, inflammatory bone loss, obesity, and diabetes, etc., suggesting therapeutic potential beyond physiological contexts. Recent evidence also shows that GIP attenuates orthodontic tooth movement by limiting mechanically induced osteoclast activity, highlighting its broader skeletal actions. In this review, we summarize recent advances regarding the role of GIP in bone metabolism, integrating evidence from cellular studies, animal models and human investigations, and discuss future directions for GIP-based interventions.

## 1. Introduction

The concept of incretin was first proposed in 1920s when intravenous (i.v.) infusion of intestinal mucosal extracts lowered blood glucose, suggesting the existence of an intestinal hormone that stimulates insulin secretion. And, the term “incretin” refers to this endocrine pancreatic secretion observed [1]. Plasma insulin levels rose much more during oral glucose administration than i.v. infusion, providing clear evidence for the existence of the “incretin effect” [2]. Two major hormones are now known to mediate this effect: glucose-dependent insulinotropic polypeptide (GIP) and glucagon-like peptide-1 (GLP-1). Both are secreted from intestinal endocrine cells in response to nutrient ingestion and act synergistically to potentiate glucose-stimulated insulin secretion, forming the core of the incretin system [3].

GIP was first purified from porcine intestinal extracts and was initially named as gastric-inhibitory polypeptide because of its ability to inhibit gastric acid secretion [4]. Subsequent studies, however, demonstrated that administration of GIP markedly potentiates insulin secretion in healthy humans and directly stimulates insulin release from pancreatic islets, firmly establishing GIP as the first incretin hormone [5]. Although GIP exerted potent insulinotropic actions at physiological concentrations, it did not meaningfully influence gastric acid secretion or gastric emptying under simulated physiological postprandial conditions. These findings led to its redefinition as glucose-dependent insulinotropic polypeptide [1,6].

**Figure 1:**
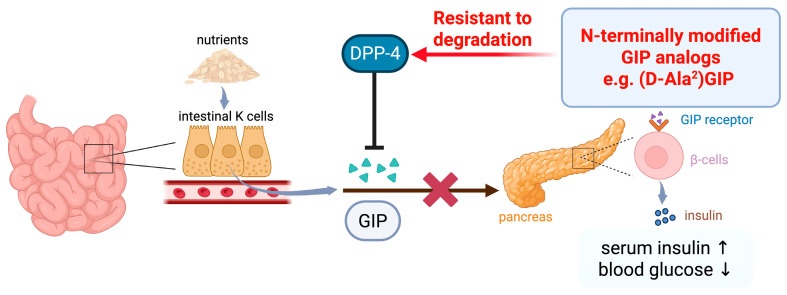
Schematic illustration of the insulinotropic function of GIP and its analogs. Nutrient intake stimulates GIP secretion from intestinal K cells. Circulating GIP binds to its receptor, GIPR, expressed on pancreatic β cells, thereby promoting glucose-dependent insulin secretion and lowering postprandial blood glucose levels. However, endogenous GIP is rapidly inactivated by DPP-4, resulting in a short circulating half-life. To overcome this limitation, N-terminally modified GIP analogs, such as (D-Ala^2^)GIP, have been developed and exhibit resistance to DPP-4–mediated degradation, thereby prolonging the biological activity of GIP and enhancing its insulinotropic effects. Arrows indicate the direction of hormone secretion and signaling. The T-shaped line denotes inhibition by DPP-4, the cross indicates reduced bioactivity of native GIP due to rapid DPP-4–mediated degradation, and upward (↑) and downward (↓) arrows indicate increased insulin secretion and decreased blood glucose levels, respectively.

GIP is now established as a 42 amino acid polypeptide, GIP (1–42), which is primarily produced and secreted from K cells located in the upper intestine [4,7]. After being secreted into the circulation, GIP binds to its receptor (GIPR), a stimulatory G protein (Gs)–coupled receptor expressed on pancreatic β cells, thereby activating adenylate cyclase and increasing intracellular cyclic adenosine monophosphate (cAMP) levels. This signaling cascade enhances glucose-dependent insulin secretion, leading to increased postprandial insulin release and reduced blood glucose after meal ingestion [3,8]. Circulating GIP (1–42) is rapidly cleaved into its inactive form, GIP (3–42), by the enzyme dipeptidyl peptidase 4 (DPP-4) in the serum, with an estimated half-life of only 5–7 min in humans [9,10]. The development of DPP-4–resistant GIP analogs has been a major strategy to prolong GIP activity, beginning from N-terminal glycation of Tyr^1^ to N-terminal substitutions, such as D-Ala^2^ or Ser^2^, which enhanced insulin secretion, and improved glucose tolerance in ob/ob mice [11,12,13,14]. A schematic overview summarizing these mechanisms is illustrated in Figure 1. Alongside GIP, GLP-1 analogs have become established antidiabetic agents due to their potent glucose-lowering effects [15,16]. Building on these successes, recent pharmacological innovations have introduced dual and triple agonists targeting GIPR, GLP-1R, and the glucagon receptor (GCGR) [17,18].

GIPR is also widely expressed in tissues outside the pancreas, including the brain, trachea, heart, gut, lungs, bone, spleen, liver, etc. [1]. Early in this century, Bollag and his colleagues detected the expression of GIPR in osteoblasts and reported the prevention of bone loss by GIP in ovariectomized mice, thereby first proposing the role of GIP in regulating bone homeostasis as part of the enteroendocrine–bone axis [19,20]. Bone resorption decreases rapidly after meal intake or oral glucose administration [21,22], adding solid evidence on this gut–bone axis. In addition to GIP, other gut hormones such as GLP-1 and glucagon-like peptide-2 (GLP-2) have also been suggested to contribute to the regulation of bone metabolism, further supporting the concept of the gut–bone axis [23]. Consistent with this concept, i.v. administration of GIP rapidly inhibited bone resorption markers in healthy humans [24], further boosted research interest in the involvement of GIP and its analogs in bone turnover.

Here in this review, we focus on the current knowledge concerning the role of GIP in physiological bone homeostasis and in bone remodeling in pathological bone conditions. We also discuss the possible mechanism of how GIP regulates bone formation and resorption. Finally, we provide insights into future direction of development and application of GIP-related medications for treating bone diseases. Schematic figures and summary tables are included to illustrate complex mechanisms and enable cross-context comparisons, thereby facilitating understanding of the topics addressed in this review.

## 2. Evidence of GIP Effects on Bone

Bone undergoes continuous remodeling through the coordinated actions of osteoclast-mediated resorption and osteoblast-mediated formation. Osteoblasts arise from mesenchymal stem cells, produce and mineralize bone matrix, and regulate osteoclast differentiation [25]. Osteoclasts originate from monocyte/macrophage lineage precursors, and their differentiation depends on macrophage colony-stimulating factor (M-CSF) and receptor activator of nuclear factor kappa-B ligand (RANKL), which is produced by osteoblasts, osteocytes, stromal cells, and activated T cells [26,27,28,29,30,31,32]. Tumor necrosis factor-α (TNF-α) also induces osteoclastogenesis, directly or via the RANKL/RANK pathway [33,34,35]. After resorbing mineralized bone, mature osteoclasts undergo apoptosis, allowing the remodeling cycle to progress to the reversal and formation phases [36]. Bone resorption demonstrates higher activity during nighttime than daytime and is further suppressed after food intake compared with fasting or i.v. glucose administration [21,22], implicating gut-derived factors in this postprandial response. Among these, GIP has emerged as a principal mediator of the “gut–bone axis,” providing a basis for the in vitro and in vivo evidence discussed below [23].

### 2.1. In Vitro Studies

#### 2.1.1. Effects of GIP on Osteoblast-Lineage Cells

GIPR is expressed in bone marrow–derived stromal or stem cells, osteoblasts, osteoclasts and osteocytes [19,37,38,39]. GIP promotes osteoblastic differentiation in bone marrow stromal cells [38]. In osteoblast-like Saos-2 cells, GIP treatment markedly enhances type I collagen expression; cell viability; alkaline phosphatase (ALP) activity; and the secretion of procollagen type I N-terminal propeptide (P1NP), a marker of bone formation, thereby demonstrating an anabolic role of GIP signaling in osteoblast function [19,40]. Moreover, GIP has been shown to inhibit apoptosis in primary human and mouse osteoblasts as well as in Saos-2 cells [41,42]. In MC3T3-E1 osteoblasts, (D-Ala^2^)GIP, a long-acting DPP-4 resistant analog, and its bone-targeting derivative (D-Ala^2^)GIP-Tag enhanced mineral deposition and promoted collagen maturation, as evidenced by increased lysyl oxidase (LOX) activity and collagen cross-linking [43,44]. The upregulation of LOX by GIP occurs through the adenylyl cyclase/cAMP/Protein Kinase A (PKA) signaling pathway, and this effect is attenuated under hyperglycemic conditions [45].

#### 2.1.2. Effects of GIP on Osteoclasts and Osteoclast Precursors

Tsukiyama et al. first reported that GIP had no inhibitory effect on the pit-forming activity of osteoclasts [41]. However, Zhong et al. later reported that GIP directly suppressed bone resorption induced by parathyroid hormone (PTH) in cultured bone explants and by RANKL in isolated osteoclasts, proposing that GIP primarily acts to inhibit active bone resorption [37]. Subsequent studies further supported this view. (D-Ala^2^)GIP dose-dependently reduced osteoclast formation and resorption in human peripheral blood mononuclear cells (PBMCs) and murine bone marrow macrophages (BMMs), through a mechanism involving suppression of intracellular Ca^2+^ rise and oscillations, which in turn decreased calcineurin activity and nuclear factor of activated T cells 1 (NFATc1) nuclear translocation [46]. In our previous study, we also observed the inhibition of (D-Ala^2^)GIP in TNF-α- and RANKL -induced osteoclastogenesis in mouse BMMs in vitro [47].

More recently, Hansen et al. showed in primary human osteoclasts that GIP directly reduces resorptive activity and induces apoptosis even in the absence of exogenous stimulators, and that these effects, including impaired NFATc1 nuclear translocation, are abolished by the selective GIPR antagonist GIP(3–30)NH_2_, indicating a GIPR-dependent intrinsic anti-resorptive action of GIP in human bone [42]. Consistently, another study also demonstrated that in primary human osteoclasts, GIP reduced NFATc1 nuclear translocation, and pre-exposure to GIP(3–30)NH_2_ prevented this effect, confirming receptor dependence of the anti-resorptive signaling [48]. Notably, cellular cAMP levels were significantly increased by GIP in human bone marrow-derived mesenchymal stem cells, Saos-2 cells, primary human osteoblasts and osteoclasts [19,39,42], suggesting that GIPR signaling contributes directly to both the promotion of bone formation and the suppression of bone resorption via cAMP-dependent mechanisms. In contrast, Mabilleau et al. reported that no cAMP increase was detected in RAW 264.7 cells, a murine macrophage-derived osteoclast precursor line [46]. This discrepancy likely reflects interspecies and differentiation-stage differences in GIPR expression or Gs-protein coupling efficiency. Functional GIPR may be expressed predominantly in mature osteoclasts, whereas precursor cells such as RAW 264.7 exhibit limited GIPR levels or reduced receptor coupling, explaining the absence of cAMP response despite evident Ca^2+^/calcineurin-dependent signaling. Further investigations are required to elucidate the molecular mechanisms by which GIP suppresses osteoclast differentiation.

Collectively, in vitro studies have established that GIP exerts dual actions on bone cells: an anabolic effect on osteoblast differentiation and survival, and an anti-resorptive effect on osteoclasts. These cellular findings consistently support a direct skeletal role of GIP signaling in maintaining bone remodeling balance through GIPR-dependent pathways. The molecular evidence of GIP-induced cAMP activation and suppression of Ca^2+^/NFATc1 signaling further highlights the complexity of its intracellular regulation. Together, these data provide mechanistic insight into how GIP contributes to the coordination of bone formation and resorption, which is further substantiated by in vivo and clinical studies discussed below.

### 2.2. Effects of Endogenous GIP Signaling on Bone

The physiological relevance of endogenous GIP signaling to bone remodeling has been investigated through genetic and pharmacological approaches. Genetic studies—ranging from engineered mouse models to analyses of naturally occurring human GIPR variants—have provided key evidence for the importance of this pathway under physiological conditions. In parallel, short-term pharmacological inhibition using the selective GIPR antagonist GIP(3–30)NH_2_ has enabled assessment of the acute skeletal effects of transiently suppressed GIP signaling in vivo. Together, these complementary strategies help delineate how physiological GIP signaling contributes to skeletal homeostasis across species.

#### 2.2.1. Genetic GIP/GIPR Alterations and Bone Phenotype in Animals and Humans

Animal studies using genetic models targeting either GIP or its receptor (GIPR) have provided important insights into the physiological role of GIP signaling in bone metabolism. Transgenic mice overexpressing GIP showed increased bone mineral density (BMD), bone mass, and bone strength, along with higher serum osteocalcin and lower pyridinoline (PYD), indicating enhanced bone formation and reduced resorption [38,49]. Conversely, global GIP-deficient mice exhibit the opposite skeletal phenotype, with reduced trabecular bone volume, decreased cortical thickness, increased osteoclast number, and lower bone formation and bone strength due to impaired matrix mineralization and collagen cross-linking [50]. Together, these gain- and loss-of-function models establish GIP as an osteotropic hormone indispensable for maintaining normal bone mass and quality through both anabolic and anti-resorptive mechanisms.

In contrast, several studies have investigated the skeletal phenotype of GIP receptor–deficient (GIPRKO) mice, and their results are not entirely consistent. Although both models demonstrated impaired bone biomechanical properties in GIPRKO mice [51,52,53], Xie et al. and Tsukiyama et al. reported reduced trabecular bone volume accompanied by increased osteoclast number and decreased bone formation [41,51]. Conversely, Gaudin-Audrain et al. observed the opposite phenotype, with increased trabecular bone volume, fewer osteoclasts, and enhanced bone formation [52]. The discrepancies among GIPRKO studies may result from differences in knockout design, metabolic compensation, and adipokine regulation. Xie and Tsukiyama deleted exons 4–5, whereas Gaudin-Audrain et al. removed exons 1–6, fully abolishing GIP binding and possibly enabling compensatory signaling such as enhanced GLP-1 sensitivity. Moreover, distinct adipokine profiles—elevated adiponectin and reduced leptin in Gaudin-Audrain’s model versus increased leptin in Xie’s—could further modulate bone remodeling through systemic metabolic pathways. Together, these animal studies highlight that disruption of GIPR signaling alters bone remodeling, although the skeletal outcome appears to depend on the knockout strategy and systemic metabolic or endocrine compensation, as summarized in Table 1.

In humans, the skeletal relevance of GIPR signaling has also been investigated through genetic association studies. In a Danish cohort study of 1686 perimenopausal women, carriers of the loss-of-function GIPR missense variant rs1800437 (E354Q) showed significantly lower BMD at the hip and femoral neck, along with a higher incidence of nonvertebral fractures [54,55]. The detrimental skeletal phenotype associated with this variant has been proposed to result from prolonged ligand–receptor interaction and increased receptor internalization, leading to desensitization of the GIP system and ultimately reduced GIPR activity in bone tissue [56]. Another large human study involving 426,824 individuals investigated two additional GIPR variants, R190Q (rs139215588) and E288G (rs143430880), both functionally impair GIPR signaling. Among these, only E288G was associated with lower BMD, while R190Q showed no detectable skeletal effect [57]. In line with these findings, a study in Swedish cohorts of young and elderly women found that the minor A allele of the GIPR variant rs10423928 was associated with lower ultrasound indices in young women, indicative of reduced bone quality, while the GIP variant rs2291725 was associated with lower femoral neck BMD and bone mineral content (BMC) as well as lower ultrasound indices in elderly women [58]. Furthermore, a Chinese study of 884 postmenopausal women reported that the T/T genotype of the same rs10423928 variant was correlated with higher femoral neck BMD, suggesting a potential bone-protective role of the T allele [59]. Together, these population-based findings imply that GIPR variants may influence bone mass and microarchitecture in an age- and ethnicity-dependent manner. More recently, a meta-analysis including up to 1.2 million individuals reported that none of the three missense variants above (E354Q, R190Q, E288G) were significantly associated with decreased BMD or increased fracture risk [60]. Importantly, this overall lack of association at the population level contrasts with the variant-specific skeletal effects observed in several individual cohort studies, supporting the notion that the impact of GIPR variants on bone may be context- and cohort-dependent rather than universal. However, the observed discrepancy between individual cohort studies and large-scale meta-analyses may arise from differences in study design, sample size, and population characteristics such as age and sex. Therefore, whether GIPR variants affect bone metabolism over the long term, or under specific systemic conditions and in particular populations, remains an intriguing question that warrants further investigation.

Taken together, animal and human genetic studies suggest that impairment of GIP or GIPR signaling can influence bone remodeling; however, the skeletal phenotype is not uniform and appears to depend on the specific gene-targeting strategy and physiological context. At present, whether these discrepancies reflect differences in local receptor function, compensatory incretin signaling, or broader metabolic changes remains unresolved and requires further investigation. To further clarify the physiological role of endogenous GIP signaling, subsequent studies have employed pharmacological approaches using selective GIPR antagonists to transiently inhibit this pathway and assess its acute effects on bone metabolism.

#### 2.2.2. GIPR Antagonist Studies

To complement the genetic evidence described above, pharmacological inhibition of GIPR signaling has been utilized to evaluate the acute skeletal effects of endogenous GIP blockade. The naturally occurring peptide GIP(3–30)NH_2_ acts as a highly selective GIPR antagonist and has been shown to counteract GIP-induced insulin secretion, adipose tissue perfusion, and triglyceride deposition [61,62]. In a human dose–response study, GIP(3–30)NH_2_ attenuated GIP’s actions in a dose-dependent manner: while 200 pmol/kg/min reduced the insulinotropic response, it did not significantly affect the GIP-induced suppression of C-terminal telopeptide of type I collagen (CTX), a marker of bone resorption; only 2000 pmol/kg/min markedly blunted the CTX reduction. This underscores that sufficient antagonist exposure is required to reveal the skeletal contribution of endogenous GIP [63]. Similarly, in a study of 10 healthy men under a 1 h 12 mmol/L hyperglycemic clamp, i.v. infusion of GIP (1.5 pmol/kg/min) reproduced postprandial plasma GIP concentrations and significantly reduced plasma CTX while increasing P1NP. Co-infusion of the selective GIPR antagonist GIP(3–30)NH_2_ (800 pmol/kg/min) markedly attenuated the CTX reduction and completely abolished the P1NP increase, demonstrating that these physiological GIP effects on bone turnover are mediated via GIPR activation [64]. Notably, GIP(3–30)NH_2_ alone did not alter bone markers under these clamp conditions. Conversely, in trials involving healthy young men and totally pancreatectomized individuals, i.v. infusion of GIP(3–30)NH_2_ alone (800 pmol/kg/min) significantly diminished the suppression of CTX induced by oral glucose tolerance test (OGTT) or liquid mixed meal tests (MMTs) compared with placebo, while no difference in P1NP levels was observed [65,66]. Because the OGTT and MMTs mimic physiological nutrient ingestion, these findings highlight the role of endogenous GIP signaling as a key mediator in the postprandial inhibition of bone resorption, independent of pancreatic hormones such as insulin, glucagon, or amylin. Although these studies appear contradictory regarding the effects of GIPR antagonism on bone turnover markers, this discrepancy primarily reflects differences in experimental context: The former tested pharmacological GIP infusion under artificial clamp conditions with minimal endogenous GIP secretion, whereas the latter assessed the physiological function of naturally secreted GIP after nutrient intake. Consistent with these observations, i.v. infusion of GIP(3–30)NH_2_ at 1200 pmol/kg/min during MMTs in type 2 diabetes patients significantly attenuated the normal postprandial CTX suppression without affecting P1NP [67], confirming that the anti-resorptive effects of endogenous GIP are preserved even under diabetic conditions. Collectively, these findings demonstrate that GIP’s bone-protective actions are GIPR-dependent and that endogenous GIP serves as a physiological mediator of postprandial inhibition of bone resorption, independent of pancreatic factors.

In contrast to human studies demonstrating a postprandial antiresorptive effect of endogenous GIP, chronic administration (3 weeks) of daily s.c. GIP(3–30)NH_2_ (25 nmol/kg) administration in rats did not alter CTX levels [68]. The lack of change in bone resorption markers may reflect the limited duration of GIPR blockade (with an estimated effective action of only ~2 h after each injection) and the natural age-related decline in CTX that could mask subtle effects, as well as inherent species differences in GIP pharmacokinetics and receptor sensitivity between rodents and humans.

Taken together, both genetic and pharmacological evidence underscores that endogenous GIP signaling plays a fundamental role in maintaining skeletal homeostasis. Human intervention studies employing the selective GIPR antagonist GIP(3–30)NH_2_ consistently demonstrate that GIP acts as a physiological inhibitor of bone resorption across different metabolic contexts—from healthy individuals to totally pancreatectomized and type 2 diabetic subjects—while its anabolic influence on bone formation appears less pronounced under physiological conditions. The concordance between these human findings and the bone phenotypes observed in GIPR-deficient animals highlights the importance of intact GIPR signaling for normal bone remodeling. Nevertheless, a combination of experimental limitations and species-specific differences in receptor pharmacodynamics, as exemplified by the lack of effect in rodent models, indicate that the translational relevance of GIP actions should be interpreted with caution. Overall, these studies collectively establish endogenous GIP as a pivotal endocrine modulator linking nutrient intake to acute suppression of bone resorption in humans.

### 2.3. Effects of Exogenous GIP Signaling on Bone in Healthy Subjects

While endogenous GIP signaling has been established as a physiological modulator of bone remodeling, its direct skeletal effects following exogenous administration remain to be clarified. To address this, both animal and human studies have examined the effects of exogenous GIP and its long-acting analogs under healthy conditions, aiming to determine whether pharmacological activation of GIPR reproduces the bone-protective actions observed with physiological signaling.

In one animal study, intraperitoneal (i.p.) administration of N-AcGIP, another long-acting DPP-4-resistant GIP analog, was conducted for 4 weeks at a dose of 25 nmol/kg/day in rats to evaluate its effects on bone in physiological conditions. The treatment had no effect in trabecular bone of the animals, but significantly improved mechanical properties of cortical bone at the macro and tissue levels, including parameters such as work to fracture, maximum load and hardness, etc. [69]. In another study, a single subcutaneous (s.c.) injection of another long-acting GIP analog designed (acylated and with an exendin-4 tail) at 1000 µg/kg in rats did not increase serum P1NP levels, but lowered CTX levels compared to the vehicle, indicating an acute inhibition effect of GIP signaling on diurnal bone resorption profile [70]. In summary, animal studies suggest that exogenous GIP predominantly suppresses bone resorption and improves cortical bone properties under physiological conditions. Although the number of animal studies is limited, these findings laid the foundation for subsequent investigations in humans, where the physiological relevance of GIP in bone metabolism has been explored more directly.

Consistent with the findings in animal models, clinical studies in healthy volunteers have demonstrated that GIP also modulates bone turnover in humans. In an early trial including 8 healthy subjects, a single i.v. bolus of 7.5 nmol GIP did not alter serum CTX levels; however, the observation period was limited to only 48 min, which likely missed the peak effect of GIP on bone resorption [71]. Subsequently, the same group conducted a more comprehensive study in 10 healthy young men. I.v. infusion of GIP (4 pmol/kg/min for 15 min followed by 2 pmol/kg/min for 45 min) significantly reduced plasma CTX levels under both euglycemic and hyperglycemic conditions, with a more pronounced suppression observed during hyperglycemia [24]. The magnitude of CTX reduction was comparable to that seen after a large meal, and circulating GIP concentrations remained within the physiological postprandial range, supporting the role of GIP as a physiological mediator of postprandial inhibition of bone resorption. These findings were further confirmed in another study involving 10 healthy male subjects. In this trial, i.v. infusion of GIP at 1.5 pmol/kg/min during a 1 h 12 mmol/L hyperglycemic clamp elicited postprandial-like plasma GIP levels, significantly suppressed serum CTX, and increased P1NP levels [64]. Notably, co-infusion of the selective GIPR antagonist GIP(3–30)NH_2_ only partially attenuated CTX suppression, possibly due to incomplete receptor blockade in bone, residual effects of hyperglycemia or insulin, or the short infusion duration. In contrast, the GIP-induced increase in P1NP was completely abolished by GIP(3–30)NH_2_, indicating that the anabolic effect on bone formation is strictly GIPR-dependent. Furthermore, s.c. administration of 200 µg GIP in 8 healthy men also reduced serum CTX and increased serum P1NP levels [72], demonstrating that peripheral GIP delivery reproduces the skeletal effects observed with i.v. and confirming its dual actions in suppressing bone resorption and promoting bone formation.

Taken together, evidence from both animal models and human studies demonstrates that exogenous GIP acutely suppresses bone resorption and, to a lesser extent, promotes bone formation under physiological conditions. These effects are observed with both i.v. and s.c. administration, at circulating GIP concentrations within the postprandial physiological range, supporting the role of GIP as a physiological regulator of postprandial bone remodeling. However, current human data are limited to small, short-term studies in healthy individuals, and the long-term skeletal consequences of GIP exposure remain unclear. Importantly, it is still unknown whether these bone-protective actions are preserved, enhanced or attenuated under pathological conditions such as estrogen deficiency, diabetes or inflammation—an issue that will be addressed in the following section.

### 2.4. Effects of Exogenous GIP Signaling on Bone Under Pathological Conditions

The effects of exogenous GIP on bone have also been investigated in pathological contexts where bone remodeling is disrupted. These conditions include estrogen deficiency–related bone loss, inflammatory bone destruction and diabetes-associated skeletal fragility, etc. Evidence from both animal models and human studies has begun to clarify how GIP signaling behaves under these stress conditions. In this section, we summarize current findings on the impact of GIP and its analogs on bone in major pathological settings.

#### 2.4.1. Postmenopausal Osteoporosis

Osteoporosis is a major bone disorder that is marked by decreased bone mass and microarchitectural deterioration, leading to increased skeletal fragility and a higher risk of fractures [73]. The pathogenesis of postmenopausal osteoporosis is largely attributed to estrogen deficiency following menopause. Loss of estrogen leads to an imbalance between bone resorption and bone formation, favoring excessive bone resorption. Mechanistically, estrogen deficiency upregulates RANKL and pro-inflammatory cytokines such as TNF-α, interleukin-1 (IL-1), and interleukin-6 (IL-6); decreases osteoprotegerin (OPG); prolongs osteoclast survival; increases sclerostin expression; and promotes osteoblast and osteocyte apoptosis [74]. The ovariectomized (OVX) rodents are widely used as an experimental model to mimic postmenopausal osteoporosis, and Bollag et al. first administered GIP in this model to investigate the effects of GIP on bone tissue [20]. The OVX rats received daily injections of GIP (0.05 mg/kg) for 6 weeks into the tail vein exhibited higher vertebral BMD compared to OVX mice receiving only vehicle. Mabilleau et al. also employed the OVX model in mice. S.c. injection of 25 nmol/kg/day N-AcGIP resulted in reduced osteoclast formation and bone resorption, as reflected by serum CTX levels, accompanied by modification of trabecular microarchitecture and improvement of biomechanical properties [46]. The same research group later developed two new GIP analogs, (D-Ala^2^)-GIP-Tag and (D-Ala^2^)-GIP_1–30_, targeting either bone or whole body GIPR, respectively. Both analogs suppressed serum CTX levels in the OVX mice, indicating reduced bone resorption. However, only daily i.p. injection of (D-Ala^2^)-GIP_1–30_ at 25 nmol/kg/day, rather than (D-Ala^2^)-GIP-Tag for 8 weeks augmented bone strength, primarily by modifying cortical microarchitecture [75]. And the absence of effect of (D-Ala^2^)-GIP-Tag in OVX mice may be due to limited bioavailability, instability after release from bone, or insufficient systemic exposure. These results further suggest that extra-skeletal GIPR activation—not only bone-localized receptors—may be necessary for GIP to exert its osteoprotective effects.

Human studies have also investigated the effects of exogenous GIP on bone metabolism in postmenopausal women, although the available evidence remains limited. In a preliminary trial involving 9 participants, a single s.c. injection of 100 µg GIP acutely suppressed nocturnal bone resorption, as reflected by reduced serum CTX levels, and increased bone formation, indicated by elevated P1NP levels [76]. To date, this remains the only human study assessing the skeletal effects of GIP administration in the postmenopausal setting. Future studies with larger sample sizes, longer infusion protocols, co-administration of DPP-4 inhibitors, or DPP-4-resistant GIP analogs are needed to determine whether more sustained and clinically meaningful effects can be achieved.

In summary, both animal and preliminary human studies provide evidence that exogenous GIP administration can suppress bone resorption, enhance bone formation, and improve trabecular as well as cortical bone microarchitecture, ultimately leading to increased bone strength. Although the precise molecular mechanisms remain to be fully elucidated, these findings highlight the therapeutic potential of GIP signaling as a strategy for the prevention or treatment of menopause-related osteoporosis.

#### 2.4.2. Inflammatory Bone Diseases

Inflammatory bone diseases are characterized by chronic inflammation-induced disruption of normal bone remodeling, resulting in excessive osteoclast activity and progressive bone loss. Representative conditions include periodontitis and rheumatoid arthritis [77]. Among them, periodontitis is a prototypical bacteria-driven inflammatory bone disorder. In this condition, lipopolysaccharide (LPS), a major component of the outer membrane of Gram-negative bacteria, recruits immune cells, stimulates the production of pro-inflammatory cytokines, and enhances osteoclastogenesis, ultimately leading to alveolar bone destruction [78,79]. Mechanistically, LPS directly upregulates RANKL expression in osteoblasts and promotes macrophages to secrete TNF-α, which in turn further increases RANKL production, synergistically amplifying osteoclast differentiation and bone resorption under inflammatory conditions [80,81,82].

To date, the role of GIP in inflammatory bone loss has been poorly characterized. Clinical studies have reported elevated GIP levels in the gingival crevicular fluid (GCF) of periodontitis patients with type 2 diabetes as well as in obese periodontitis patients without diabetes [83,84]. Serum GIP concentrations are also higher in patients with rheumatoid arthritis [85]; however, whether these elevations exert any functional influence on local bone metabolism remains unclear. Moreover, no studies to date have explored GIP signaling in other inflammatory bone diseases, such as osteomyelitis.

A previous study reported that GIPRKO mice exhibited increased inflammatory cell infiltration and elevated inflammatory gene expression in the gingiva tissue during periodontitis, suggesting a potential anti-inflammatory role of GIP in periodontal tissues [86]. Although these findings did not directly assess bone remodeling, inflammatory microenvironments are known to promote osteoclast activation, raising the possibility that impaired GIP signaling may indirectly contribute to inflammatory bone loss. In parallel, no study had previously tested whether pharmacological activation of GIP signaling could counteract inflammatory bone loss. Our group was the first to address this question using a mouse calvarial osteolysis model, in which LPS was injected subcutaneously for 5 consecutive days to induce inflammatory osteolysis, with or without (D-Ala^2^)GIP co-administration (25 nmol/kg/day) [47]. (D-Ala^2^)GIP treatment significantly reduced osteoclast formation in calvaria sutures, suppressed tartrate-resistant acid phosphatase (TRAP) and cathepsin K (CTSK) expression in bone tissue, and markedly diminished bone resorption on micro-CT analysis. In parallel, LPS-induced TNF-α and RANKL expression in calvaria bone was attenuated by GIP treatment. In vitro, (D-Ala^2^)GIP similarly inhibited LPS-induced TNF-α expression in macrophages and RANKL production in osteoblasts via dampening LPS-triggered mitogen-activated protein kinase (MAPK) pathway, suggesting that GIP suppresses inflammatory osteoclastogenesis through inhibition of pro-inflammatory cytokine signaling.

Although these results were obtained from a short-term acute inflammatory model, they provide the first experimental evidence that exogenous GIP signaling can protect bone under inflammatory stress. This suggests that GIP may exert immunomodulatory effects beyond its classic incretin function and may serve as a potential therapeutic target for inflammation-related osteolysis.

#### 2.4.3. Obesity and Diabetes

Obesity and diabetes mellitus are major metabolic disorders that profoundly affect bone homeostasis. Both have a high global prevalence, with over 600 million people affected by obesity and an estimated 1.3 billion projected to have diabetes by 2050 [87,88]. Diabetes mellitus includes type 1 (T1DM) and type 2 (T2DM); both feature chronic hyperglycemia, but T1DM results from autoimmune destruction of pancreatic β cells and absolute insulin deficiency, whereas T2DM arises from insulin resistance and relative insulin deficiency [89]. Obesity contributes to both insulin resistance and β cell dysfunction, predisposing individuals to prediabetes and T2DM. Importantly, obesity and T2DM share overlapping mechanisms—adipose tissue inflammation, ectopic lipid deposition, and multi-organ insulin resistance—indicating that T2DM represents an advanced stage of obesity-related metabolic dysfunction [90].

T1DM and T2DM display distinct skeletal phenotypes. T1DM is characterized by low bone mass and impaired bone formation due to insulin deficiency, reduced insulin-like growth factor 1 (IGF-1) signaling, and accumulation of advanced glycation end-products (AGEs) that deteriorate bone matrix quality. In contrast, T2DM often shows normal or increased BMD but reduced turnover and inferior material quality, linked to chronic hyperinsulinemia, inflammation, and marrow adiposity. Despite these differences, both types share an elevated fracture risk, highlighting bone quality rather than quantity as the key determinant of diabetic skeletal fragility [91,92]. Obesity similarly increases BMD through mechanical loading and estrogen, yet compromises bone quality via marrow fat accumulation and inflammatory adipokines, producing a “dense-yet-brittle” phenotype comparable to that seen in T2DM [93,94].

An animal study investigated the effects of GIP analogs on bone under obese conditions. In a diet-induced obese mouse model, 42 consecutive days of i.p. injections with (D-Ala^2^)GIP or its bone-targeting derivative (D-Ala^2^)-GIP-Tag (25 nmol/kg/day) enhanced femoral mechanical strength—reflected by increased ultimate bending load and work-to-fracture—without altering trabecular microarchitecture, indicating that the improvement likely resulted from enhanced collagen maturity and matrix composition [44]. In humans, i.v. co-infusion of GIP (4 pmol/kg/min) and glucose, administered to establish an isoglycemic intravenous glucose infusion (IIGI) condition, in 17 overweight or obese men caused a transient, non-significant rise in plasma P1NP but markedly reduced plasma CTX levels compared with baseline and isoglycemic OGTT conditions, suggesting a strong antiresorptive effect of GIP that is at least partially independent of insulin [95].

Both animal and human studies have examined the influences of GIP analogs on bone in diabetes mellitus. In a single-dose streptozotocin (STZ, 150 mg/kg)–induced diabetic mouse model mimicking human T1DM, daily i.p. administration of (D-Ala^2^)GIP (25 nmol/kg) for 21 days did not prevent reductions in whole-bone mechanical properties, such as ultimate load and stiffness, nor did it rescue cortical microstructure deterioration. However, it significantly improved mechanical performance at the tissue level, including maximum force and hardness, and markedly enhanced the collagen integrity index, indicating improved bone matrix quality. Moreover, (D-Ala^2^)GIP showed a mild, non-significant tendency to preserve bone volume fraction (BV/TV) and trabecular number (Tb.N). It effectively prevented diabetes-induced reductions in dynamic bone formation indices (MAR, MS/BS, BFR/BS) and osteoclast parameters (N.Oc/B.Pm, Oc.S/BS), collectively suggesting partial restoration of bone remodeling despite limited structural effects [96]. In a human study involving 10 C-peptide–negative male patients with T1DM, plasma P1NP and CTX were measured under low- or high-glycemic clamp conditions maintained by insulin or glucose infusion, respectively, during i.v. GIP infusion (4 pmol/kg/min). GIP transiently increased plasma P1NP compared with saline at low glycemia, but the difference disappeared at 90 min. In contrast, plasma CTX was markedly reduced, suggesting that GIP suppresses bone resorption independently of glucose or insulin levels [97]. Additionally, another study including 20 men with T1DM investigated the effects of a 6-day continuous s.c. infusion of GIP (6 pmol/kg/min). Serum CTX levels were significantly decreased during the initial 3 h of infusion; however, this difference was no longer significant after 1 and 6 days. P1NP concentrations showed a transient, non-significant upward trend [98]. This remains the only study so far examining the prolonged effects of GIP infusion on bone turnover markers in patients with diabetes, suggesting a possible tachyphylaxis of bone responsiveness following sustained exogenous GIP exposure, which warrants further investigation.

In the context of T2DM, no animal study has yet evaluated the effects of exogenous GIP or its analogs alone on bone metabolism. In human studies, a single s.c. injection of 200 µg GIP rapidly suppressed serum CTX levels and increased P1NP levels in 10 men with T2DM, to the same extent of that observed previously in healthy individuals [99]. Meanwhile, another trial involving 12 T2DM male patients examined the effects of i.v. GIP infusion (4 pmol/kg/min for 15 min followed by 2 pmol/kg/min for 75 min) under three different glycemic conditions: insulin-induced hypoglycemia, fasting hyperglycemia, and aggravated hyperglycemia. Plasma CTX concentrations were consistently reduced by GIP under all glycemic conditions, whereas P1NP levels showed only a transient increase under hypoglycemia and no sustained differences from saline [100]. In another study including 9 individuals with T2DM, i.v. co-infusion of GIP with glucose to establish an IIGI condition (4 pmol/kg/min for 20 min followed by 2 pmol/kg/min for 30 min) similarly reduced plasma CTX concentrations without altering P1NP levels [101]. Collectively, these findings indicate that although the insulinotropic effects of GIP are blunted in patients with T2DM, GIP retains its inhibitory effect on bone resorption.

Collectively, the available evidence demonstrates that GIP exerts consistent antiresorptive effects across obese and diabetic conditions, while its influence on bone formation remains subtle and transient. In both T1DM and T2DM, GIP infusion markedly decreases circulating CTX levels regardless of glycemic state, suggesting a direct inhibitory action on osteoclast activity that is largely independent of insulin. However, the stimulation of bone formation markers such as P1NP appears weak, short-lived, and statistically nonsignificant, implying that the anabolic potential of GIP is limited under diabetic conditions. Animal studies further indicate that (D-Ala^2^)GIP improves bone matrix quality and mechanical strength even without increasing bone mass, suggesting that its skeletal benefits primarily derive from matrix-level regulation rather than changes in bone quantity. Taken together, these findings indicate that while the insulinotropic effects of GIP are blunted in metabolic disease, its skeletal antiresorptive action is preserved. The long-term consequences of sustained GIP receptor activation, including the potential development of tachyphylaxis, remain to be clarified through extended experimental and clinical studies.

#### 2.4.4. Other Pathological Conditions

Beyond the major bone disorders discussed above, several human studies have also examined the effects of GIP in other pathological conditions with bone involvement. In one study including four female patients with postsurgical hypoparathyroidism, s.c. injection of 100 μg GIP markedly reduced plasma CTX levels despite very low or undetectable PTH concentrations, indicating that its antiresorptive effect does not rely on PTH-mediated pathways. Plasma P1NP levels showed a non-significant increase. The study had a small sample size, and some participants were taking concomitant medications that might have influenced bone turnover; however, the inclusion of a placebo control helped mitigate this potential bias. These findings suggest that GIP can modulate bone turnover even under severely impaired PTH signaling, warranting further confirmation [102]. In another study of 25 adults with pancreatic-insufficient cystic fibrosis (PI-CF), i.v. GIP infusion (4 pmol/kg/min for 80 min) acutely reduced plasma CTX levels after 30 min, suggesting that the bone anti-resorptive effect of GIP is retained in PI-CF, even though its insulinotropic action is blunted in this population [103]. This finding supports the concept that GIP can directly regulate bone remodeling independently of insulin. Collectively, these findings highlight the therapeutic potential of targeting GIP signaling to preserve bone mass in conditions such as hypoparathyroidism and cystic fibrosis. Moreover, exogenous GIP or its analogs may confer protective effects in other metabolic disorders accompanied by bone deterioration—such as chronic kidney disease or Cushing’s syndrome—which merits further investigation.

These observations, together with evidence from postmenopausal osteoporosis, inflammatory bone diseases, and metabolic disorders such as obesity and diabetes described in earlier sections, indicate that exogenous GIP modulates bone turnover across a broad spectrum of pathological contexts. Detailed in vivo animal studies evaluating exogenous GIP are summarized in Table 2, and human studies are summarized separately in Table 3. A concise visual comparison of endogenous versus exogenous GIP actions across physiological and pathological conditions is provided in Table 4.

### 2.5. Effects of GIP Signaling on Orthodontic Tooth Movement

Beyond pathological bone conditions, GIP signaling also influences mechanically driven bone remodeling. Orthodontic tooth movement (OTM) is a mechanically induced form of bone remodeling, during which orthodontic force triggers coordinated changes in the periodontal ligament and alveolar bone. Tooth movement is achieved through osteoclastic bone resorption on the compression side and osteoblastic bone formation on the tension side [104,105]. Osteocytes, the most abundant cells in bone, function as key mechanosensors that convert mechanical loading into biochemical signals and coordinate spatial–temporal bone remodeling during OTM [106]. OTM is accelerated under conditions that enhance osteoclastogenesis, such as ovariectomy, hypertension, and micro-osteoperforation [107,108,109]. Cytokines produced locally in the periodontal environment play central roles in regulating osteoclast formation during OTM, including TNF-α, M-CSF, RANKL, IL-1, IL-2, IL-6, OPG, etc. [110]. Among these, TNF-α is a major pro-inflammatory mediator that promotes osteoclastogenesis and thereby facilitates tooth movement. TNF-α expression was detected on the compression side during OTM [111], and both OTM distance and osteoclast numbers were reduced in TNF receptor–deficient mice [112]. Moreover, impaired TNF-α–induced osteoclast formation contributed to diminished OTM in aged mice [113]. TNF-α further induces RANKL and sclerostin expression and triggers osteocyte necroptosis, collectively promoting osteoclastogenesis during OTM [114,115,116]. These findings highlight TNF-α as a key molecular driver of force-induced bone remodeling.

Our group previously demonstrated that pharmacological agents used for diabetes management can influence OTM. Local gingival injection of the DPP-4 inhibitor linagliptin (30 μg every 2 days) significantly reduced tooth movement distance and osteoclast formation on the compression side compared with phosphate-buffered saline (PBS) controls [117]. Similarly, local administration of the GLP-1R agonist exendin-4 (20 μg) attenuated OTM and osteoclastogenesis [118]. In both models, expression of TNF-α and RANKL in periodontal tissues was markedly lower than in controls, suggesting that the suppressed OTM likely resulted from direct inhibition of TNF-α– or RANKL-driven osteoclastogenesis. These findings underscore the need for careful orthodontic management in diabetic patients receiving incretin-based medications.

Evidence on the role of GIP signaling in OTM remains sparse. In a previous study, GIPRKO mice exhibited significantly increased OTM distance and osteoclast formation on the compression side relative to wildtype controls [119], suggesting that similar to GLP-1 signaling, GIP signaling exerts an inhibitory influence on mechanical-force–driven bone remodeling. Consistent with this, we previously demonstrated that (D-Ala^2^)GIP suppressed TNF-α– and RANKL-induced osteoclastogenesis in vitro [47]. Although direct assessment of exogenous GIP administration in OTM models has not yet been reported, these findings raise the possibility that local delivery of GIP might similarly dampen osteoclast formation and attenuate tooth movement.

## 3. Future Perspectives and Conclusions

**Figure 2:**
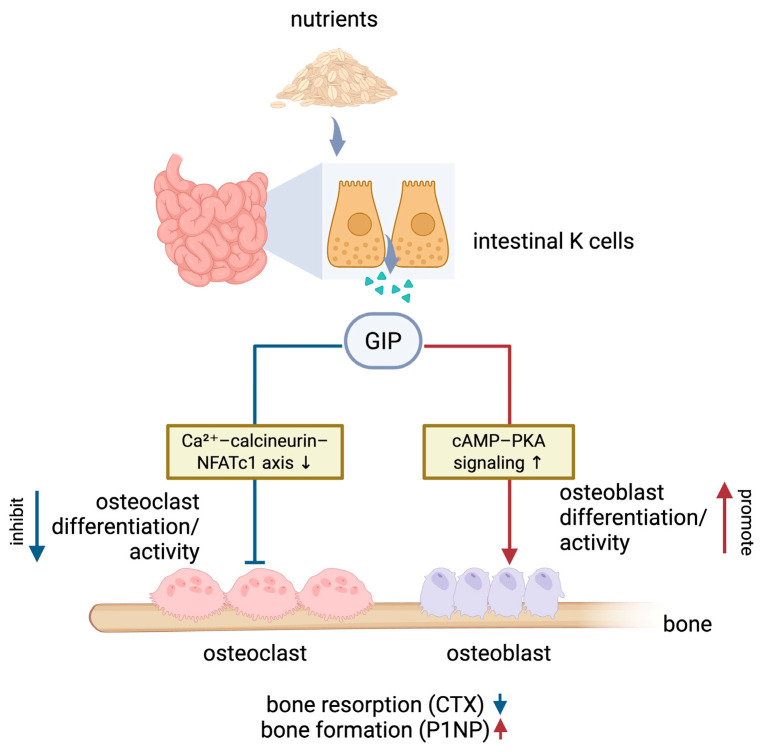
Schematic illustration of the physiological actions of GIP on bone. Following nutrient ingestion, GIP is secreted from intestinal K cells and exerts dual regulatory effects on bone remodeling. In osteoclasts, GIP suppresses differentiation and resorptive activity, at least in part through inhibition of the Ca^2+^–calcineurin–NFATc1 signaling axis. In contrast, GIP promotes osteoblast differentiation and activity via activation of cAMP–PKA signaling. Together, these coordinated actions lead to reduced bone resorption, reflected by decreased circulating CTX levels, and enhanced bone formation, as indicated by increased circulating P1NP. Arrowed lines indicate promoting effects, whereas the T-shaped line denotes inhibition. The symbols ↑ and ↓ denote increased or decreased signaling pathway activity, cellular differentiation or function, or circulating bone turnover markers, respectively, as indicated.

In recent years, our understanding of the endocrine crosstalk between the gut and the skeleton has expanded substantially, particularly regarding GIP, laying important foundation for therapeutic exploration. In this review, we summarized current insights into the role of the incretin hormone GIP in skeletal biology across in vitro, animal, and human studies. Physiologically, GIP suppresses postprandial bone resorption, and genetic studies indicate that intact GIP signaling is required to maintain normal bone mass and quality. To provide an integrated overview of these physiological actions, a schematic representation of postprandial GIP secretion and its regulatory effects on osteoblast and osteoclast activity, highlighting the underlying intracellular signaling pathways, is shown (Figure 2). In pathological states such as estrogen deficiency, obesity and diabetes, and inflammatory osteolysis, exogenous GIP consistently demonstrates anti-resorptive actions, supporting the rationale for developing GIP-based therapeutic strategies for skeletal disorders. Nevertheless, most available studies have relied on short-term administration, capturing only acute responses. Given that bone remodeling is a slow, continuous process, future work is needed to determine whether the protective skeletal effects of GIP can be maintained long-term. In addition, GIP signaling warrants broader investigation across a wider range of skeletal disorders beyond the disease contexts discussed above. Future studies should clarify how GIP interacts with these distinct pathological environments and delineate the specific cellular and molecular pathways engaged by GIP signaling. Such insights will be essential to clarify the scope of GIP-based interventions and to determine where GIP-targeted strategies may offer distinct therapeutic value.

For decades, the impaired insulinotropic response to GIP in patients with T2DM limited research and drug development [1,3]. Recently, however, several dual and triple multi-receptor incretin agonists incorporating GIPR activation have been developed, including combinations targeting GIPR with GLP-1R or GLP-2R, as well as tri-agonists engaging GIPR, GLP-1R, and GCGR. Co-administration of GIP with GLP-2, another gut hormone known to suppress bone resorption, additively reduced CTX in postmenopausal women [76], and a unimolecular dual GIP/GLP-2 analog improved bone strength and microarchitecture in OVX mice [120]. Concomitant i.v. infusion of GIP, GLP-1, and GLP-2 also lowered CTX to levels comparable to OGTT responses [101]. In addition, a triple agonist targeting GIPR, GLP-1R, and GCGR, (D-Ala^2^)GIP–oxyntomodulin, enhanced trabecular microarchitecture and bone strength in a leptin receptor–deficient diabetic mouse model [121]. Furthermore, tirzepatide, a dual GIPR/GLP-1R agonist has recently been approved for treatment of diabetes and obesity [122,123]. Emerging animal and clinical studies report its mixed skeletal outcomes, ranging from largely neutral effects on bone parameters to reports of increased bone loss or fracture risk in individuals with diabetes or obesity. Although evidence remains limited, these concerning findings have been attributed primarily to weight reduction, while the precise mechanisms remain unclear [124,125,126,127].

**Figure 3:**
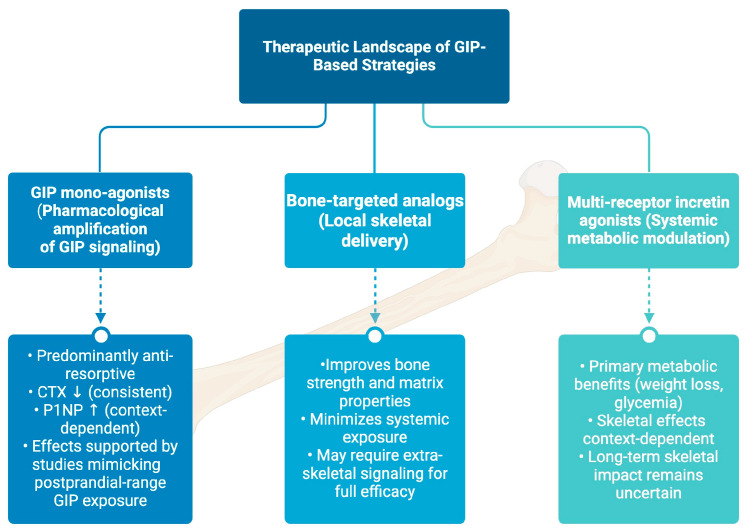
Therapeutic landscape of GIP-based strategies in metabolic bone diseases. GIP mono-agonist strategies, including native GIP administration and pharmacological GIP analogs, predominantly exert anti-resorptive effects, consistently reducing bone resorption marker CTX, while effects on bone formation markers P1NP appear context-dependent. These skeletal effects are supported by experimental and clinical studies employing GIP exposure levels within the postprandial range. Bone-targeted GIP analogs are designed to enhance local skeletal delivery, improving bone strength and matrix properties while minimizing systemic metabolic exposure; however, full efficacy may still require coordinated extra-skeletal signaling. In contrast, multi-receptor incretin agonists, such as tirzepatide, primarily provide systemic metabolic benefits, including weight loss and improved glycemic control, with skeletal outcomes that vary depending on metabolic context and remain incompletely defined over long-term treatment. This schematic illustration integrates current evidence and highlights distinct therapeutic paradigms through which GIP-based interventions may influence bone metabolism in the context of metabolic bone diseases. The arrows ↑ and ↓ indicate increases or decreases in bone turnover markers, respectively.

To place the emerging GIP-based pharmacological strategies discussed in the present review into a broader therapeutic context, a conceptual schematic is provided (Figure 3). This diagram illustrates how GIP mono-agonists, bone-targeted GIP analogs, and multi-receptor incretin agonists—including tirzepatide—may differentially fit into current and potential treatment paradigms for metabolic bone diseases, while highlighting the complexity and context-dependence of their skeletal effects.

Overall, targeting GIP signaling appears to be a promising approach for the treatment of pathological bone loss. However, more systematic and long-term investigations across diverse skeletal disease contexts are required to fully establish its therapeutic relevance. In parallel, multi-receptor agonists that incorporate GIPR activation represent an additional promising direction, with the potential to enhance skeletal outcomes while retaining the metabolic and insulinotropic benefits essential for patients with diabetes or obesity.

## Figures and Tables

**Table 1:** Summary of GIPR knockout strategies, skeletal phenotypes, and proposed explanations for discrepant findings in mice.

GIPR Knockout Strategies	Reference	Skeletal Phenotypes	Proposed Explanations for Discrepancies
Exons 4–5 deletion	[41,51]	Biomechanical properties ↓ Trabecular bone volume ↓ Bone formation ↓ Osteoclast number ↑	Lesser extent of GIPR deletion; Adipokine profile difference—leptin ↑
Exons 1–6 deletion	[52]	Biomechanical properties ↓ Trabecular bone volume ↑ Bone formation ↑ Osteoclast number ↓	Greater extent of GIPR deletion—GLP-1 signaling compensation; Adipokine profile difference—adiponectin ↑ & leptin ↓

↑: increased relative to control; ↓: decreased relative to control.

**Table 2:** Summary of in vivo animal studies evaluating the effects of exogenous GIP on bone across pathological conditions.

Pathological Model	Treatment Dose	Administration	Duration	Bone Effect	Reference
OVX rats	GIP 0.05 mg/kg	i.v.	6 weeks	Vertebral BMD ↑	[20]
OVX mice	N-AcGIP 25 nmol/kg	s.c.	Not reported	Osteoclast formation ↓ Bone resorption ↓ Biomechanical properties ↑ Modified trabecular microarchitecture	[46]
OVX mice	(D-Ala^2^)-GIP_1-30_ 25 nmol/kg	i.p.	8 weeks	Bone resorption ↓ Bone strength ↑ Modified cortical microarchitecture;	[75]
	(D-Ala^2^)-GIP-Tag 25 nmol/kg			Bone resorption ↓	
LPS-induced bone inflammation	(D-Ala^2^)GIP 25 nmol/kg	s.c.	5 days	Osteoclast formation ↓ Bone resorption ↓	[47]
Diet-induced obesity	(D-Ala^2^)GIP 25 nmol/kg	i.p.	6 weeks	Bone strength ↑ No effects on bone microarchitecture	[44]
	(D-Ala^2^)-GIP-Tag 25 nmol/kg				
STZ-induced T1DM mice	(D-Ala^2^)GIP 25 nmol/kg	i.p.	3 weeks	Tissue-level bone strength ↑ Restored bone remodeling No effects on cortical microarchitecture	[96]

↑: increased relative to control; ↓: decreased relative to control.

**Table 3:** Summary of human studies investigating the effects of exogenous GIP on bone under pathological conditions.

Pathological Model	Treatment Dose (GIP)	Administration	Duration	Bone Effect	Reference
Postmenopausal women	100 μg	s.c.	Single bolus	CTX ↓ P1NP ↑	[76]
Overweight/ Obesity	4 pmol/kg/min IIGI	i.v.	4 h	CTX ↓ P1NP ↔	[95]
T1DM	4 pmol/kg/min Low/high glycemic clamp	i.v.	90 min	CTX ↓ P1NP ↑ (transient, low glycemia)	[97]
	6 pmol/kg/min	s.c.	6 days	CTX ↓ (3 h) P1NP ↔	[98]
T2DM	200 μg	s.c.	Single bolus	CTX ↓ P1NP ↑	[99]
	4 → 2 pmol/kg/min varied glycemic conditions	i.v.	15 → 75 min	CTX ↓ P1NP ↑ (transient, hypoglycemia)	[100]
	4 → 2 pmol/kg/min IIGI	i.v.	20 → 30 min	CTX ↓ P1NP ↔	[101]
Hypoparathyroidism	100 μg	s.c.	Single bolus	CTX ↓ P1NP ↔	[102]
PI-CF	4 pmol/kg/min	i.v.	80 min	CTX ↓	[103]

↑: increased relative to control or baseline; ↓: decreased relative to control or baseline; ↔: no significant change relative to control or baseline.

**Table 4:** Visual summary of skeletal actions of endogenous versus exogenous GIP across health and major pathological conditions.

Context	Endogenous GIP (Genetic/Antagonist Studies)	Exogenous GIP (GIP/GIP Analog Studies)
Health	Postprandial bone resorption ↓ Maintaining skeletal homeostasis	Bone resorption ↓; formation ± ↑ Cortical bone properties ↑
Postmenopausal osteoporosis (OVX/postmenopausal women)	—	Bone resorption ↓; formation ↑ Bone strength/microarchitecture ↑
Inflammation	Potential protective effect	Bone resorption ↓
T1DM	—	Restored bone remodeling Tissue-level bone strength ↑
T2DM	Postprandial bone resorption ↓	Bone resorption ↓; formation ± ↑

↑: increased relative to control or baseline; ↓: decreased relative to control or baseline; ±: context-dependent responses.

## Data Availability

Data are available from the corresponding authors upon reasonable request.

## Abbreviations

The following abbreviations are used in this manuscript:

I.v.	Intravenous
GIP	Glucose-dependent insulinotropic polypeptide
GLP-1	Glucagon-like peptide-1
GIPR	Glucose-dependent insulinotropic polypeptide receptor
Gs	Stimulatory G protein
cAMP	Cyclic adenosine monophosphate
DPP-4	Dipeptidyl peptidase-4
GLP-1R	Glucagon-like peptide-1 receptor
GCGR	Glucagon receptor
GLP-2	Glucagon-like peptide-2
M-CSF	Macrophage colony-stimulating factor
RANKL	Receptor activator of nuclear factor kappa-B ligand
TNF-α	Tumor necrosis factor-α
RANK	Receptor activator of nuclear factor kappa-B
ALP	Alkaline phosphatase
P1NP	Procollagen type I N-terminal propeptide
LOX	Lysyl oxidase
PKA	Protein Kinase A
PTH	Parathyroid hormone
PBMCs	Peripheral blood mononuclear cells
BMMs	Bone marrow macrophages
NFATc1	Nuclear factor of activated T cells 1
BMD	Bone mineral density
PYD	Pyridinoline
GIPRKO	GIP receptor-deficient
BMC	Bone mineral content
CTX	C-terminal telopeptide of type I collagen
OGTT	Oral glucose tolerance test
MMTs	Mixed meal tests
I.p.	Intraperitoneal
S.c.	Subcutaneous
IL-1	Interleukin-1
IL-6	Interleukin-6
OPG	Osteoprotegerin
OVX	Ovariectomy
LPS	Lipopolysaccharide
GCF	Gingival crevicular fluid
TRAP	Tartrate-resistant acid phosphatase
CTSK	Cathepsin K
MAPK	Mitogen-activated protein kinase
T1DM	Type 1 diabetes mellitus
T2DM	Type 2 diabetes mellitus
IGF-1	Insulin-like growth factor 1
AGEs	Advanced glycation end-products
IIGI	Isoglycemic intravenous glucose infusion
STZ	Streptozotocin
BV/TV	Bone volume fraction
Tb.N	Trabecular number
MAR	Mineral apposition rate
MS/BS	Mineralizing surface per bone surface
BFR/BS	Bone formation rate per bone surface
N.Oc/B.Pm	Number of osteoclasts per bone perimeter
Oc.S/BS	Osteoclast surface per bone surface
PI-CF	Pancreatic-insufficient cystic fibrosis
OTM	Orthodontic tooth movement
PBS	Phosphate-buffered saline
GLP-2R	Glucagon-like peptide-2 receptor
